# The pulmonary toxicity of carboxylated or aminated multi-walled carbon nanotubes in mice is determined by the prior purification method

**DOI:** 10.1186/s12989-020-00390-y

**Published:** 2020-11-26

**Authors:** Alexia J. Taylor-Just, Mark D. Ihrie, Katherine S. Duke, Ho Young Lee, Dorothy J. You, Salik Hussain, Vamsi K. Kodali, Christina Ziemann, Otto Creutzenberg, Adriana Vulpoi, Flaviu Turcu, Monica Potara, Milica Todea, Sybille van den Brule, Dominique Lison, James C. Bonner

**Affiliations:** 1 Toxicology Program, Department of Biological Sciences, North Carolina State University, 850 Main Campus Drive, Suite 1104, Toxicology Building, Raleigh, NC 27606 USA; 2 Department of Physiology and Pharmacology, School of Medicine, West Virginia University, Morgantown, WV USA; 3 Fraunhofer Institute for Toxicology and Experimental Medicine ITEM, Hannover, Germany; 4 Interdisciplinary Research Institute in Bio-Nano-Sciences, Babes Bolyai University, Cluj-Napoca, Romania; 5 Department of Molecular Sciences, Faculty of Medicine, Iuliu Hatieganu University of Medicine and Pharmacy, Cluj-Napoca, Romania; 6 Louvain centre for Toxicology and Applied Pharmacology (LTAP), Institut de Recherche Expérimentale et Clinique (IREC), Université catholique de Louvain (UCL), Brussels, Belgium

**Keywords:** Carbon nanotubes, Purification, Functionalization, Lung injury, Fibrosis

## Abstract

**Background:**

Inhalation of multi-walled carbon nanotubes (MWCNTs) poses a potential risk to human health. In order to safeguard workers and consumers, the toxic properties of MWCNTs need to be identified. Functionalization has been shown to either decrease or increase MWCNT-related pulmonary injury, depending on the type of modification. We, therefore, investigated both acute and chronic pulmonary toxicity of a library of MWCNTs derived from a common pristine parent compound (NC7000).

**Methods:**

MWCNTs were thermally or chemically purified and subsequently surface functionalized by carboxylation or amination. To evaluate pulmonary toxicity, male C57BL6 mice were dosed via oropharyngeal aspiration with either 1.6 or 4 mg/kg of each MWCNT type. Mitsui-7 MWCNT was used as a positive control. Necropsy was performed at days 3 and 60 post-exposure to collect bronchoalveolar lavage fluid (BALF) and lungs.

**Results:**

At day 3 all MWCNTs increased the number of neutrophils in BALF. Chemical purification had a greater effect on pro-inflammatory cytokines (IL-1β, IL-6, CXCL1) in BALF, while thermal purification had a greater effect on pro-fibrotic cytokines (CCL2, OPN, TGF-β1). At day 60, thermally purified, carboxylated MWCNTs had the strongest effect on lymphocyte numbers in BALF. Thermally purified MWCNTs caused the greatest increase in LDH and total protein in BALF. Furthermore, the thermally purified and carboxyl- or amine-functionalized MWCNTs caused the greatest number of granulomatous lesions in the lungs. The physicochemical characteristics mainly associated with increased toxicity of the thermally purified derivatives were decreased surface defects and decreased amorphous content as indicated by Raman spectroscopy.

**Conclusions:**

These data demonstrate that the purification method is an important determinant of lung toxicity induced by carboxyl- and amine-functionalized MWCNTs.

**Supplementary Information:**

The online version contains supplementary material available at 10.1186/s12989-020-00390-y.

## Introduction

With advancements in nanotechnology, the application of carbon nanotubes (CNTs) in commercial products has been increasing since their discovery in 1991 [1]. Both single-walled (SWCNTs) and multi-walled carbon nanotubes (MWCNTs) have emerged as important classes of engineered nanomaterials. Their outstanding stability, tensile strength, electric conductivity, and thermal resistance [2, 3] have prompted their use in various industrial applications, including composite materials, nanoelectronics, field-effect emitters, and hydrogen storage [4]. However, the potential technical benefits of CNTs in these applications are hindered by publications reporting pulmonary toxicity and pathogenicity in experimental animal models [5]. While occupational exposure to CNTs has already been documented [6–12], the forecasted increase in CNT production, development of functionalized variants, and expansion of novel applications all indicate an inevitable increase in human and environmental exposures to CNTs. Therefore, it is of utmost importance to identify the hazardous properties and toxicity mechanisms of CNTs to implement “safer-by-design” concepts.

Identifying the impact of specific physicochemical characteristics of MWCNTs that contribute to toxicity is imperative in ensuring safe application of highly variable forms of MWCNTs. Some of the physicochemical properties of MWCNTs that have previously been linked to pulmonary inflammation and fibrosis include length, diameter, rigidity, surface charge, and the presence of residual metal catalysts from the manufacturing process [13, 14]. The generation of oxidative stress also seems to play an important role in MWCNT-induced toxicity [15]. Notably, the similarity in the fiber-like structure of MWCNTs with that of asbestos or other bio-persistent fibers suggests similar pathogenic effects such as the development of lung fibrosis or induction of mesothelioma [16–20]. CNTs have been shown to cause pulmonary inflammation, fibrosis and cancer when administered to the lungs of mice and rats [21, 22]. Lung fibrosis is exaggerated in conjunction with allergens such as house dust mite or ovalbumin [23–26].

A diversity of CNTs are currently obtained by different synthesis methods to yield products with varying concentric layers (single-, double-, and multi-walled), as well as CNT with different length, diameter and purity [27]. In addition, a wide variety of post-synthesis treatments are used to modify the surface chemistry of CNTs, including carboxylation, amination, and surface coating with inorganic or organic thin films [28]. In vivo studies with rodents as well as cell culture experiments have found that post-synthesis treatment of CNTs has an impact on their pro-inflammatory and fibrotic potential. Exposure of mice to nitrogen doped MWCNTs resulted in less pulmonary inflammation when compared to untreated MWCNTs [29]. Some studies found that carboxylated MWCNTs induced less pulmonary inflammation and fibrosis in mice as compared to pristine MWCNTs [30–32]. Contrastingly, acid functionalization of SWCNTs seemed to increase pulmonary toxicity in mice [33]. Coating MWCNTs by atomic layer deposition (ALD) with zinc oxide increases the severity of the acute pulmonary inflammatory response in mice [34], while aluminum oxide, applied by ALD, resulted in less pulmonary fibrosis [35]. Carboxyl and hydroxyl functionalization of MWCNTs was shown to increase genotoxicity but to reduce cell death in an in vitro model, using a human lung epithelial cell line [36]. The diverse experimental data (in vitro cell culture, intratracheal instillation, oropharyngeal aspiration, inhalation, peritoneal injection, intra-pleural injection) do not indicate a consistent toxicological profile associated with MWCNT modifications. Consequently, it is not possible to group these materials into similar entities that can be assessed collectively for their hazardous properties.

As part of the ERA-NET SIINN project ICONS (“International Collaboration on Nanotube Safety”), we examined the consequences of purification, followed by functionalization, on MWCNT-induced inflammation and fibrosis in the lungs of mice. All purified and functionalized MWCNTs were derived from a single batch of Nanocyl NC7000 that was previously tested in vivo [37, 38]. The goal of this investigation was to provide knowledge on the hazardous properties of MWCNTs by assessing the relationship between purification and functionalization of MWCNTs on lung toxicity. Male C57BL/6 mice were chosen based on their common use in toxicology studies and were exposed by oropharyngeal aspiration, a surrogate method for inhalation. We hypothesized that the prior purification method would influence the pulmonary toxicity of amine or carboxyl functionalized MWCNTs. The experimental design included a panel of endpoints (inflammatory cell counts, LDH, total protein, cytokines, and pathology) that were examined at 3 days post-exposure for acute inflammation and 60 days post-exposure for chronic fibrosis. The toxicity data were evaluated in the context of a robust physicochemical characterization of the MWCNTs.

## Results

### Characterization of the MWCNT library

**Fig. 1:**
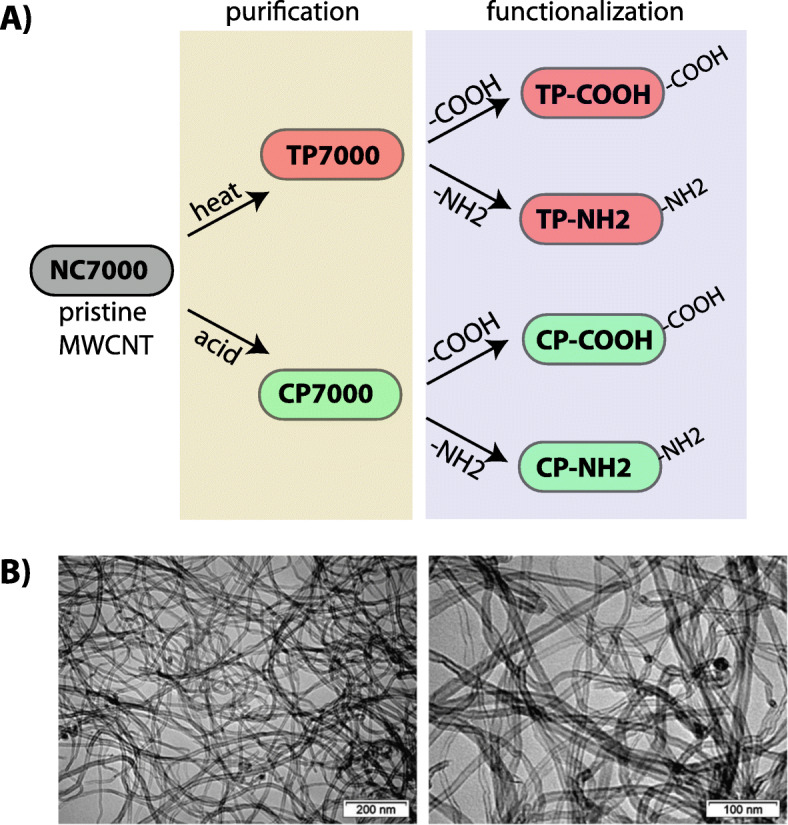
Library of MWCNTs used in the present study. **a** Illustration showing derivation of carboxyl and amine functionalized MWCNTs after purification by heat or acid treatment. **b** TEM images of the NC7000 MWCNT samples. Magnification bars are indicated

The custom synthesized library of MWCNTs shown in Fig. 1a was thoroughly characterized as described in the *Methods and Materials* section. This library of purified and functionalized MWCNTs was generated from a parent, pristine MWCNT termed NC7000. Representative TEM images of NC7000 are shown in Fig. 1b. TEM analysis demonstrated that NC7000 had a mean diameter of 12 nm and an average length of 1350 nm. EDX analysis demonstrated that NC7000 was composed of ~ 92% carbon, ~ 3% oxygen, 4.5% Al and 0.27% transition metals (Fe and Co) (Table 1). ICP-MS demonstrated similar results for metal residues in NC7000; 4.4% Al and 0.54% transition metals (Co and Fe) (Table 1). Thermal or chemical purification of NC7000 to yield TP7000 or CP7000 respectively, removed > 95% of Al and > 50% of transition metals (Co and Fe) from the parent NC7000 MWCNT sample (Table 1). Raman spectroscopy was further employed to evaluate the structural properties of MWCNT samples as this technique provides specific information about the graphitic materials in terms of the defects and ordering in the structure [39, 40]. The Raman spectra of NC7000, TP7000, TP-COOH, TP-NH2, CP7000, CP-COOH and CP-NH2 samples in the region of 900–2100 cm^− 1^ featuring the characteristic bands of MWCNTs are shown in Additional File 1. For instance, the band at ~ 1570 cm^− 1^ (G band) is connected to the graphitic nature of the sample (i.e., crystallinity of the sample, pristine arrangement of atoms), while the band at ~ 1346 cm^− 1^ (D mode) is indicative to the presence of defects (i.e. carbonaceous impurities with sp^3^ bonding, broken sp^2^ bonds in the side wall) [41]. A deconvolution procedure was performed on the spectra to reveal the fractions of the G and D bands (Additional File 2). Five Raman bands were identified, located at around 1340 (D1 band -associated to the disordered graphitic lattice, corresponding to a graphitic lattice vibration mode with A1g symmetry), 1570 (G band - connected to ideal graphitic lattice (E2g-symmetry), 1610 (D2 band- related to disordered graphitic lattice, corresponding to a graphitic lattice mode with E2g symmetry), 1460 (D3 band – connected to the presence of amorphous carbon) and 1200 cm^− 1^ (D4 band – related to disordered graphitic lattice (A1g symmetry) and ionic impurities [39] (Additional File 2). The parameters obtained by deconvolution of the Raman spectroscopy data are shown in Additional File 3. The acquired Raman parameters were used to estimate the structural changes of MWCNTs induced by thermal and chemical purification. For instance, the ratio between the intensities of D1 and G bands (ID1/IG) is a good indicator of disorder/defect evolution in MWCNTs. As illustrated in Additional File 3 and summarized in Table 1, thermal purification of NC7000 to yield TP7000 reduced the ID1/IG ratio by > 50%, indicating that the thermal purification method was indeed effective in decreasing defects in the MWCNTs. The Kaiser test confirmed that nitrogen was present on both amine functionalized MWCNTs, i.e. TP-NH2 and CP-NH2 (Table 1). XPS analysis pointed to an increase in oxygen content for both carboxyl functionalized MWCNTs, i.e. TP-COOH and CP-COOH (Table 1). Also, Additional File 4 shows XPS C1s high resolution spectra with the peak positions of the possible bonds*.* In addition, BET and pore volume analysis showed that amine and carboxyl functionalized MWCNTs exhibit 3 to 6 times higher specific surface area, as compared to their purified TP7000 and CP7000 parent MWCNTs, and 3 to 5 times higher pore volume (Table 1). The hydrodynamic size distribution of the different MWCNT types in dispersion medium (0.1% Pluronic solution in sterile DPBS + 3% BSA) used for delivery to the lungs of mice was assessed by dynamic light scattering (DLS) and surface charge was measured by zeta potential (Table 2). DLS demonstrated that NC7000 agglomerates ranged from 259 to 6152 nm in diameter after ultrasonication in dispersion medium. Thermal or chemical purification increased the average diameter 3 to 4-fold, while carboxylation reduced the diameter similar to NC7000 and amination did not change the diameter compared to the purified parent compounds. DLS measurements indicated a broad size distribution in the dispersed mixtures, with polydispersity index (pDi) ranging from 0.46 to 1. The pDi distribution indicated that the NC7000 MWCNT sample was the least heterogeneous suspension (pDi = 0.57) while thermally or chemically purified MWCNTs (TP7000 and CP7000, respectively) were the most heterogeneous suspensions (pDi = 1.0). All MWCNT samples had no detectable levels of endotoxin (< 0.05 U/ml, as cut-off criterion), as determined by the Limulus amoebocyte lysate test (data not shown).

**Table 1:** Physicochemical characteristics of the MWCNT library

MWCNT sample	ICP-MS^a^ residues (%)		Raman spectroscopy		EDX^b^			XPS^c^			Kaiser test	BET^d^	Pore volume
	Al	TE^e^	ID1/IG	AC^f^	C (%)	O(%)	N(%)	C(%)	O(%)	N(%)	(μmol/g)	(m^2^/g)	(μl/g)
**NC7000**	4.43	0.54	1.29	4.68	92.05	3.19	0	97.8	1.4	0	–	24	61
**TP7000**	0.04	0.16	0.47	0.48	98.89	1.06	0	99.4	0.6	0	–	30	83
**TP-COOH**	–	–	0.41	0.81	98.59	1.22	0	95.1	4.4	0.5	–	141	381
**TP-NH2**	–	–	0.39	1.92	90.97	5.99	3.02	86.3	7.3	6.4	47	182	402
**CP7000**	0.13	0.15	1.28	4.07	98.88	0.98	0	98.1	1.9	0	–	30	111
**CP-COOH**	–	–	1.16	5.00	95.47	4.33	0	94.1	5.9	0	–	179	521
**CP-NH2**	–	–	1.28	4.53	97.62	1.17	0.78	98.6	0.7	0.5	62	111	330

^a^TE, transition elements (Co and Fe)

^b^EDX, energy dispersive X-ray analysis

^c^XPS, X-ray photoelectron spectroscopy

^d^BET, Brunauer, Emmett and Teller analysis

^e^TE, transition elements (Co and Fe)

^f^AC amorphous carbon content estimated from the area of the D3 band expressed as area percent from the total deconvoluted Raman spectra (see Additional File 3)

**Table 2:** Suspension characteristics of MWCNTs in dispersion medium^a^used for oropharyngeal aspiration in mice

MWCNT	Size distribution^b^		pDi	Zeta potential (mV)
	Z-Avg Diameter nm	Multi-mode distribution peaks Diameter nm (% peak area)		
NC7000	399 ± 6	259 ± 36 (55%) 1652 ± 1194 (45%)	0.57	−16.8 ± 1.4
TP7000	1626 ± 265	683 ± 205 (82%) 193 ± 91 (19%)	1	− 14.6 ± 1.1
TP-COOH	932 ± 49	716 ± 337 (83%) 174 ± 39 (17%)	0.83	−15.7 ± 1.4
TP-NH2	1590 ± 114	992 ± 214 (75%) 267 ± 39 (25%)	0.97	− 18.0 ± 0.4
CP7000	1359 ± 169	719 ± 403 (60%) 200 ± 12 (40%)	1	−15.3 ± 1.4
CP-COOH	484 ± 48	637 ± 145 (57%) 153 ± 29 (43%)	0.72	− 24.0 ± 0.9
CP-NH2	1897 ± 216	918 ± 85 (100%)	0.80	−17.6 ± 1.2
Mitsui-7	985 ± 73	919 ± 227 (92%) 3292 ± 2685 (8%)	0.46	−14.8 ± 0.8

^a^MWCNTs were diluted in dispersion medium (0.1% Pluronic solution in sterile DPBS + 3% BSA) to a concentration of 50 μg/ml prior to DLS analysis

^b^Values are mean ± standard deviation of 4–5 independent measurements. Multimode distribution is presented as diameter of different peaks and % area occupied by that peak (relative contribution)

### Thermally purified, carboxyl functionalized MWCNTs caused enhanced acute and chronic inflammatory cell responses in the lungs of mice

**Fig. 2:**
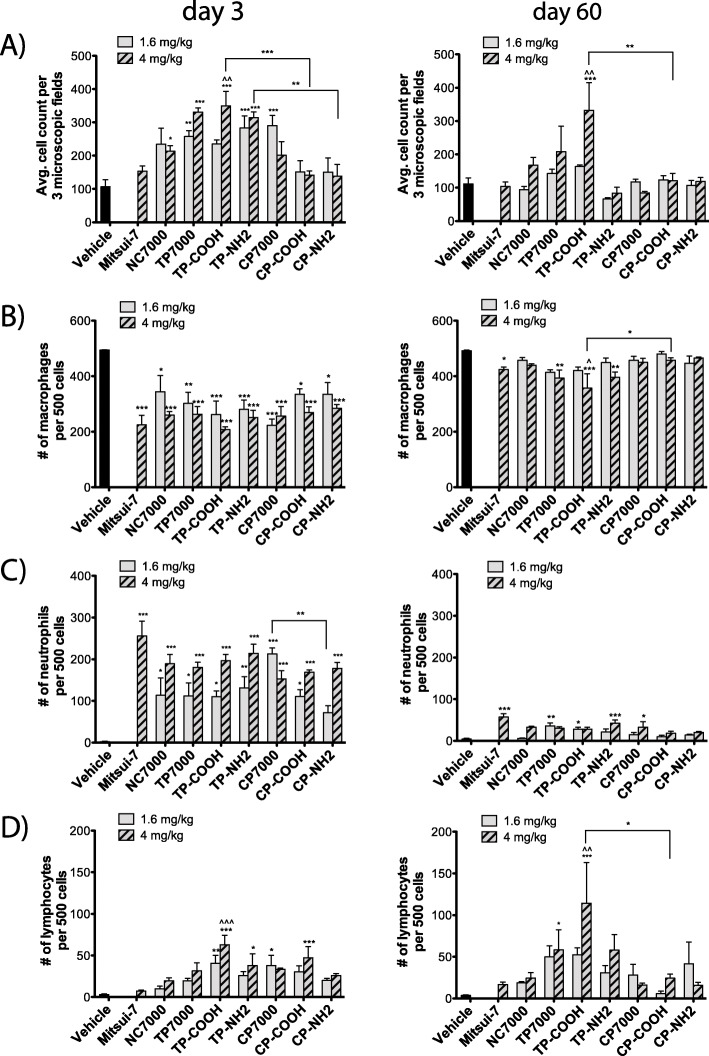
Inflammatory cell total counts and differentials in BAL fluid at 3 or 60 days after exposure to a low or high dose of purified and functionalized MWCNTs. **a** Average cell counts (based on 3 representative photomicrographs). **b** Macrophages (per 500 cells). **c** Neutrophils (per 500 cells). **d** Lymphocytes (per 500 cells). Statistically significant difference to the vehicle control: *** *P* < 0.001, ** *P* < 0.01, * *P* < 0.05 or to the NC7000 parent MWCNT sample: ^^^ *P* < 0.001, ^^ *P* < 0.01, ^ *P* < 0.05

Mice were exposed to a low (1.6 mg/kg) and a high (4 mg/kg) dose of the library of MWCNTs via oropharyngeal aspiration in a 0.1% pluronic/3% BSA saline solution as the vehicle. The high dose of 4 mg/kg has been used previously for MWCNTs delivered to the lungs of mice by oropharyngeal aspiration [21, 26]. Mitsui-7 MWCNTs were used as the positive control. Negative control mice were exposed to the vehicle solution alone. At days 3 and 60 post-exposure, the mice were euthanized via i.p. pentobarbital injection followed by collection of bronchoalveolar lavage fluid (BALF) from the lungs. At 3 days, exposure to all different MWCNTs, except Mitsui-7, CP-COOH and CP-NH2 MWCNTs, resulted in a significant increase in the number of inflammatory cells in BALF. TP-COOH induced significantly higher cell counts when compared to pristine NC7000 and CP-COOH. There was also a significant difference between the two aminated MWCNTs, with TP-NH2 inducing higher total cell counts as compared to CP-NH2. (Fig. 2a). At 60 days, only TP-COOH at the higher dose (4 mg/kg) caused a significant increase in total BALF inflammatory cells, even when compared to NC7000 and CP-COOH. Cell differentials indicated that macrophages were no longer dominant in the BALF 3 days after MWCNT exposure, but were back closer to normal numbers by day 60 (Fig. 2b). Neutrophils were increased in the BALF with all types of MWCNTs at 3 days, especially at the higher dose. The CP-NH2-exposed animals exhibited a significantly lower number of neutrophils in BALF than CP7000-exposed mice (Fig. 2c). By 60 days, there was still an elevated number of neutrophils in BALF from mice, treated with Mitsui-7, NC7000, TP7000, CP7000 and TP-NH2, but to a much lesser extent, as compared to day 3 post-exposure. Lymphocytes were significantly increased in BALF by TP-COOH, TP-NH2, CP7000 and CP-COOH at 3 days, especially at the higher dose (Fig. 2d). The largest increase in lymphocytes at day 3 post-exposure was observed for TP-COOH and CP-COOH, with TP-COOH showing significantly increased lymphocyte numbers when compared to NC7000. By 60 days, the number of lymphocytes in BALF doubled with a sustained significant increase after TP-COOH exposure as well as after TP7000 administration. Numbers of lymphocytes, due to TP-COOH exposure were also significantly increased, when compared to the parent NC7000 or the chemically purified and carboxylated CP-COOH. Overall, the TP-COOH MWCNT sample had the greatest effect on both the acute and chronic lung inflammatory cell responses.

### The purification method seems to determine the potency of MWCNTs to cause chronic lung injury in mice

**Fig. 3:**
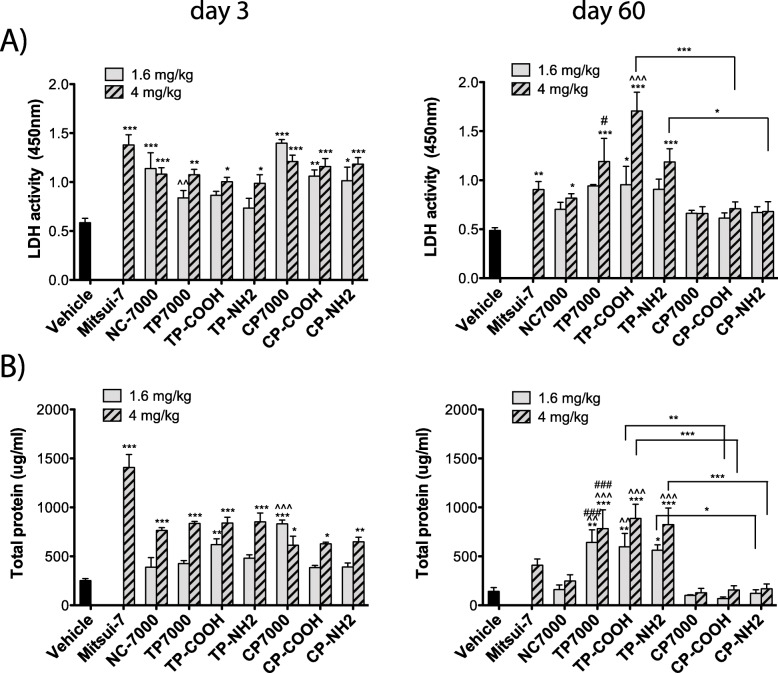
Lactate dehydrogenase (LDH) activity and total protein in BAL fluid at 3 or 60 days after exposure to a low and high dose of purified and functionalized MWCNTs. **a** LDH activity. **b** Total protein. Statistically significant difference to the vehicle control: *** *P* < 0.001, ** *P* < 0.01, * *P* < 0.05, the parent NC7000 sample: ^^^ *P* < 0.001, ^^ *P* < 0.01, or to the chemically-purified sample CP7000: ### *P* < 0.001, # *P* < 0.05

Lung injury was assessed by measuring lactate dehydrogenase (LDH) enzyme activity in BALF, collected from mice exposed to the different MWCNTs. At 3 days, all different MWCNT types induced a statistically significant increase in LDH activity, at the higher dose (4 mg/kg). At the lower dose, there was a significant induction in LDH activity by the pristine NC7000 and the chemically purified CP7000 samples (Fig. 3a). At 60 days, LDH levels in BALF remained significantly elevated in mice treated with the higher MWCNT dose of Mitsui-7, and the pristine and thermally purified MWCNT samples only. There was no marked increase for the three chemically purified MWCNTs, irrespective of the surface functionalization (Fig. 3a). At 60 days post-exposure, there was a significant difference in LDH activity between the functionalized MWCNTs when based on purification method; the TP-COOH sample caused greater LDH activity compared to the CP-COOH sample, and the TP-NH2 sample caused greater LDH activity compared to the CP-NH2 sample. A similar trend was observed, when measuring amounts of total protein in the BALF (Fig. 3b). At 3 days, total protein in BALF was increased by exposure to the higher dose of all different MWCNT types when compared to the vehicle control. Protein levels were still significantly enhanced at day 60 post-exposure (both low and high dose) by the three thermally purified MWCNT samples. There was also a significant difference between the functionalized MWCNTs when comparing based on purification method, with thermal purification resulting in more protein in the BALF. A lung damaging effect was noted for both the chemically purified samples and pristine NC7000.

### The purification method seems to alter MWCNT-induced pro-inflammatory and pro-fibrotic cytokine levels in the lungs of mice

**Fig. 4:**
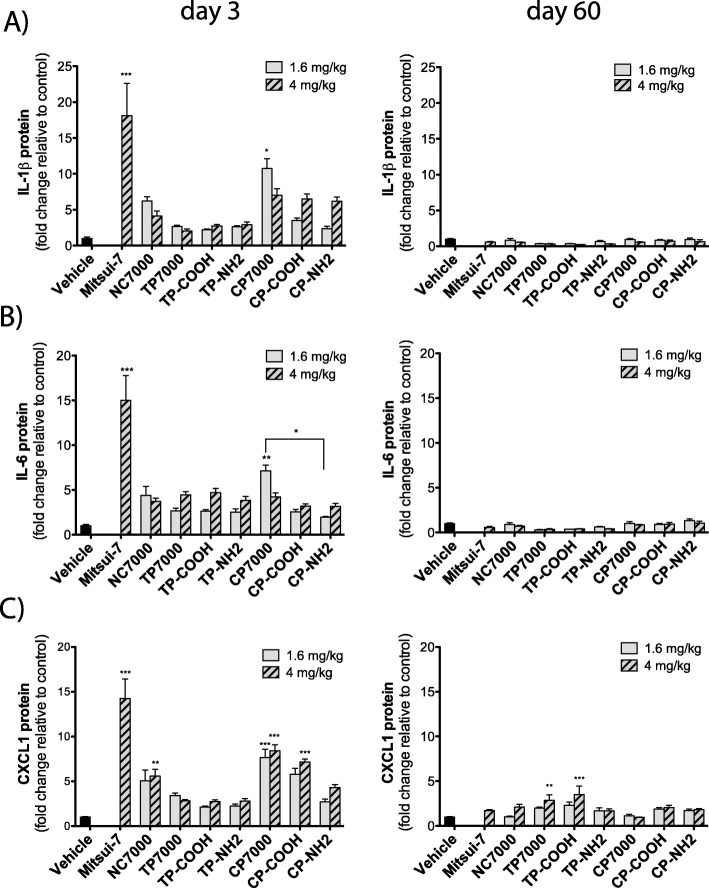
Pro-inflammatory cytokines in the BAL fluid at 3 or 60 days after exposure to a low and high dose of purified and functionalized MWCNTs measured by ELISA. **a** IL-1β. **b** IL-6. **c** CXCL1. Statistically significant difference to the vehicle control: *** *P* < 0.001, ** *P* < 0.01, * *P* < 0.05, or between CP7000 and CP-NH2 (* *P* < 0.05) as indicated by the bar

**Fig. 5:**
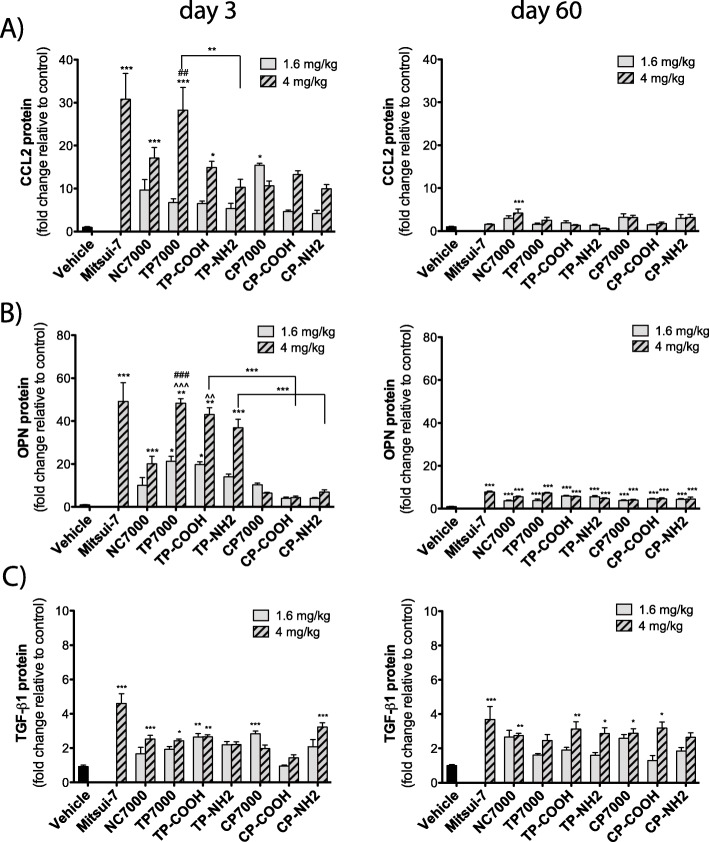
Pro-fibrotic cytokines in the BAL fluid at 3 or 60 days after exposure to a low and high dose of purified and functionalized MWCNTs measured by ELISA. **a** CCL2. **b** OPN. **c** TGF-β1. Statistically significant difference to the vehicle control: *** *P* < 0.001, ** *P* < 0.01, * *P* < 0.05, the parent NC7000 MWCNT sample: ^^^ *P* < 0.001, ^^ *P* < 0.01, or the chemically-purified CP7000 sample: ### *P* < 0.001, ## *P* < 0.01

Secreted pro-inflammatory cytokine protein levels (IL-1β, IL-6, CXCL1) were measured by ELISA in BALF of mice following exposure to the different MWCNT types. At day 3 post-exposure, besides the positive control Mitsui-7, only the lower dose of CP7000 caused a statistically significant increase in IL-1β (10-fold) and IL-6 (7-fold) protein levels, as compared to vehicle control animals (Fig. 4a & b). Protein levels of the neutrophil chemokine CXCL1 were significantly elevated 3-days after exposure to the higher dose of the pristine NC7000 sample (5-fold), the chemically purified CP7000 sample (9-fold) and the chemically purified and carboxyl-functionalized CP-COOH sample (7-fold) (Fig. 4c). By 60 days, the protein levels for these cytokines returned to control levels for IL-1β and IL-6. For CXCL1 only, a statistically significant increase in secreted protein level at day 60 post-exposure was noted with the higher dose of TP7000 (3-fold) and TP-COOH (4-fold). The secreted protein levels of the monocyte chemoattractant CCL2 were also measured in the BALF (Fig. 5a). The higher dose of TP7000 at day 3 post-exposure resulted in the highest statistically significant increase (27-fold) in CCL2 secretion of all MWCNTs tested, but a statistically significant elevation was also present for the high dose of NC7000 (16-fold) and TP-COOH (15-fold) and the low dose of CP7000 (15-fold). TP-NH2 exposure resulted in a lower level of secreted CCL2, when compared to the TP7000 sample. By 60 days, CCL2 levels had returned nearly to control levels, except for the higher dose of the parent NC7000 sample (4-fold). Protein levels of the secreted pro-fibrotic cytokines OPN and TGF-β1 were also measured in BALF. At 3 days, OPN levels were significantly elevated after exposure to the group of thermally purified MWCNTs (40 to 50-fold), but not to the chemically purified MWCNT samples (Fig. 5b). OPN levels were also significantly elevated (20-fold) with the parent NC7000 sample, but to a lesser degree than with the thermally purified MWCNTs. There was also a significant difference between the functionalized MWCNTs when the comparison was based on the purification method, with thermal purification resulting in more OPN protein in BALF than chemical purification of NC7000. At day 60 post-exposure, OPN levels were increased by all MWCNT types, irrespective of the purification method used or the type of surface functionalization, but to a minor degree (5 to 10-fold), as compared to day 3 post-exposure. TGF-β1 protein levels were significantly increased by all MWCNT samples at 3 days post-exposure, except for TP-NH2, CP7000 and CP-COOH samples (Fig. 5c). At the lower dose (1.6 mg/kg), only TP-NH2 and CP7000 mediated a statistically significant elevation in TGF-β1 protein levels. A statistically significant induction of TGF-β1 secretion was also observed for all MWCNT samples, except for TP7000 and CP-NH2, at day 60 post-exposure, when animals were exposed to 4 mg/kg of the materials.

### Lung granulomatous lesions were induced by thermally purified, and subsequently functionalized MWCNTs

**Fig. 6:**
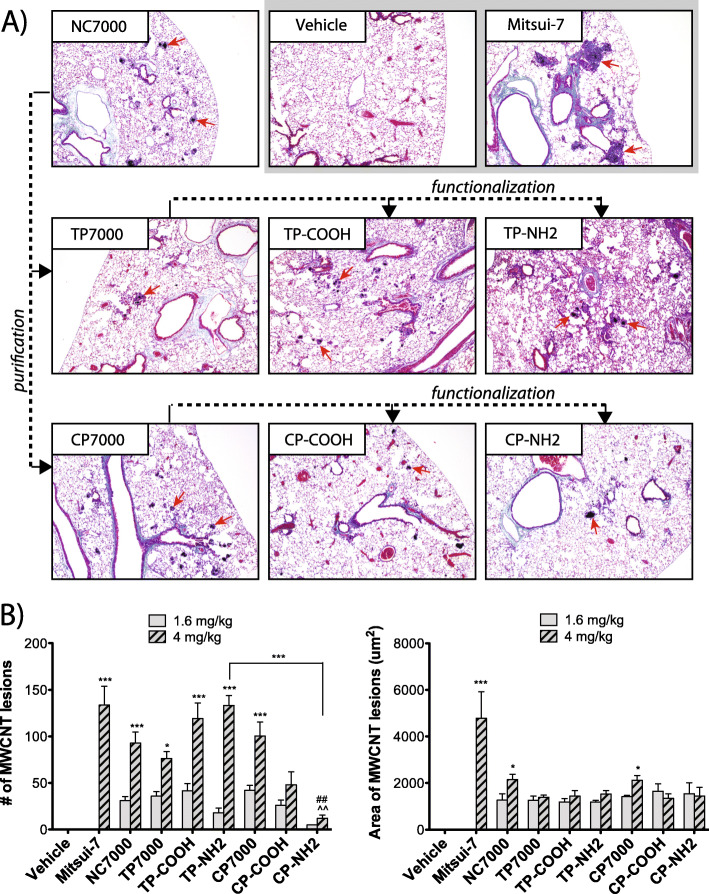
Histopathology at 60 days after exposure to a high dose of purified and subsequently functionalized MWCNTs. **a** Representative photomicrographs taken at 40x magnification of Gomori’s trichrome-stained lung sections. Red arrows indicate granulomatous lesions in lung tissue. Dotted lines with arrows linking panels indicate derivation of the purified or functionalized MWCNTs with the parent pristine NC7000 MWCNT sample. **b** Results of quantitative morphometry showing numbers of granulomatous lesions (left graph) and size (area) of granulomatous lesions (right graph). *N* = 4 mice per group/treatment. Statistically significant difference to the vehicle control: *P < 0.05, ***P < 0.001, or to the chemically purified CP7000 sample: ## *P* < 0.01 compared to CP7000

Histopathologic analysis was used to determine if a chronic 60-day exposure to the library of MWCNTs results in granulomatous lesions in the mouse lung. Lung sections were visualized using Gomori’s trichrome stain with collagen staining blue (Fig. 6a). Granulomatous lesions containing MWCNTs in the lung sections were counted and the area quantified by morphometry. The vehicle control lungs contained a normal amount of collagen and no granulomatous lesions, while the lungs of mice in the Mitsui-7 positive control group contained a significant number of large granulomatous lesions (average area: 4783.5 ± 1132.4 μm^2^, average number per mouse: 133.6 ± 20.4). Exposure to the higher dose (4 mg/kg) of nearly all MWCNT species resulted in a significantly increased number of granulomatous lesions in the lung (Fig. 6b). The lower dose (1.6 mg/kg) produced some granulomatous lesions, but not to a statistically significant extent. The greatest number of granulomatous lesions was induced by the 4 mg/kg dose of the carboxylated TP-COOH (average number per mouse: 119.25 ± 16.6) and the aminated TP-NH2 MWCNT samples (average number per mouse: 133.25 ± 10.7). CP-COOH and CP-NH2 were the only MWCNTs that did not result in a statistically significant number of granulomatous lesions, as compared to vehicle control. Furthermore, we observed a significant difference between the functionalized MWCNTs, when comparison was based on the purification method used, with thermal purification resulting in more granulomatous lesions. The lower number of lesions produced by CP-NH2 exposure was also statistically significant when compared to NC7000 and CP7000 MWCNTs (Fig. 6b). The area of the granulomatous lesions was significantly increased by the positive control, Mitsui-7. Smaller albeit significant increases in lesion size were induced by the parent MWCNT NC7000 and the chemically purified MWCNT CP7000.

## Discussion

In the present study, we sought to determine if the purification method prior to functionalization of MWCNTs might influence the acute and chronic pulmonary responses of mice in vivo. To our knowledge, this is the first study to evaluate pulmonary inflammatory and fibrotic responses to a library of functionalized (carboxylation and amination) MWCNTs generated by two different purification methods (thermal vs chemical) prior to surface functionalization. Notably, all MWCNT species were derived from a common pristine parent MWCNT sample (NC7000). The key finding of this study was that thermal purification of MWCNTs followed by carboxyl or amine functionalization caused a greater increase in pro-fibrotic cytokines in BALF (OPN, CCL2) at day 3 post-exposure and more severe chronic lung injury (LDH and total protein in BALF) with increased number of granulomatous lesions in the lungs of mice at day 60 post-exposure. In contrast, chemical purification caused a greater acute increase in pro-inflammatory cytokines in BALF (IL-1β, IL-6, CXCL1) at day 3 post-exposure, but did not significantly increase LDH, total protein or OPN in BALF at 60 days. Furthermore, chemical purification did not increase numbers of granulomatous lesions in the lungs of mice. The parent MWCNT sample, NC7000, caused a relatively weak increase in these biomarkers of lung injury and caused an intermediate chronic fibrotic response. The major physicochemical features associated with the increased pulmonary toxicity of thermally purified MWCNTs was a reduction in surface defects and amorphous carbon content, both revealed by Raman spectroscopy (discussed in more detail below).

Functionalization is used to make CNTs more compatible for various applications (e.g., increased solubility and dispersion in aqueous media), but inconsistent data on toxicity and limited control over the behavior of functionalized CNTs currently restrict predictability of such applications [42]. By comparing a series of functionalized SWCNTs in vitro, Sayes et al. (2006) found that the cytotoxic activity towards human fibroblasts decreased with the density of functionalization [43]. Roda et al. (2011) compared the toxicity of pristine and functionalized MWCNTs (amine and carboxylic groups) 16 days after intratracheal instillation in rats, using a dose (0.2 mg/rat or ~ 1 mg/kg), comparable to the low dose of MWCNTs used in the present study (1.6 mg/kg) [44]. Inflammatory responses were reported, irrespective of functionalization, but no fibrosis was detected. Carboxyl functionalization has also been reported to increase the bioactivity of MWCNTs in macrophages [45]. However, we previously reported that carboxylated MWCNTs had reduced pro-inflammatory activity in the lungs of mice, compared to pristine MWCNTs, as determined by neutrophil counts in BALF 24 h after oropharyngeal aspiration [46]. Others have also reported that COOH-functionalization of MWCNTs reduced their fibrotic activity in mice [47, 48]. Furthermore, Li et al. (2013) reported that anionic functionalization (carboxyl or PEG) reduced the pro-inflammatory activity of MWCNTs in vitro as well as the lung fibrotic response in mice [32]. Functionalization of long CNTs used by Poland et al. (2008) with an ammonium-terminated tri(ethylene glycol) chain reduced inflammation in the peritoneal cavity of mice, likely due to disaggregation of individual CNTs [49, 50]. These studies collectively demonstrate that surface functionalization with carboxyl or amine groups can increase or decrease toxicity, depending on a number of variables, including the specific types of CNTs used, the functionalization protocols employed, and the biological systems studied.

Characterization of our library of MWCNTs (Table 1) indicated that functionalization increases the specific surface area of the MWCNTs. BET surface area and pore volume were also higher in the functionalized MWCNTs, regardless of the purification method. Our XPS data show that the MWCNTs functionalized by carboxylation after either thermal purification (TP-COOH) or chemical purification (CP-COOH) were indeed functionalized with carboxyl groups. Amine functionalization of MWCNTs after thermal purification (TP-NH2) or chemical purification (CP-NH2) was confirmed by the Kaiser test. The ID1/IG ratio (i.e., the ratio between the intensities of D1 and G bands obtained by Raman spectroscopy that are indicative of surface defects) can be used to obtain information regarding structural changes caused by functionalization [51]. However, in our study functionalization with carboxyl or amine groups did not increase the ID1/IG ratio of thermally or chemically purified MWCNTs, which might indicate that functionalization was relatively mild (Table 1). On the other hand, others have shown that changes in the ID1/IG ratio are generally seen after functionalization of single- or double-walled CNTs, while changes in the ID1/IG ratio for MWCNTs are much smaller or nonexistent [52, 53]. Raman spectroscopy also showed that thermal purification caused a greater reduction in amorphous carbonaceous content compared to chemical purification (Table 1, Additional Files 1, 2, 3 and 4). Amorphous carbon adsorbed on the sidewalls of CNTs can contribute to the D band and alter the ID1/IG ratio [51]. Thus, it is possible that the reduction in amorphous carbon after thermal purification could be one of the sources of the ambiguous changes observed. To overcome this and confirm effective functionalization, we have performed X-ray photoelectron spectroscopy (XPS) on the surface of the tubes. The efficacy of functionalization can be clearly accessed from the XPS data (Table 1) which shows an increase in oxygen and nitrogen peaks from the COOH and NH_2_ functionalizations, respectively, and various chemical bonds from the functional groups (Additional File 4).

Our results suggest that functionalization alone is not the important driver of toxicity, but rather the purification method used prior to functionalization. In the present study, Raman spectroscopy showed a reduced ID1/IG ratio for the thermally purified MWCNT derivatives (Table 1, Additional Files 1, 2, 3 and 4), which also had greater pulmonary toxicity compared to the chemically purified MWCNT derivatives. The ID1/IG ratio is an indicator of defects in MWCNTs. Our data suggest that decreased defects caused by thermal purification could be a reason for the greater toxicity (e.g., LDH and total protein) as well as increased chronic granuloma formation. Defects or imperfections in the carbon framework of CNTs have been suggested to play a role in the toxicological properties of CNTs. For example, Muller et al. (2008) reported that structural defects in MWCNTs were responsible for their acute lung toxicity as well as for genotoxic effects, but not for the long-term fibrotic responses [54]. This is seemingly in contrast to our findings. However, there were some notable differences between that study and the present study. For example, Muller et al. used MWCNTs that were disrupted by grinding and were then subsequently modified by heating, whereas we began with pristine MWCNTs (NC7000). It is possible that grinding MWCNTs increases structural defects. In addition, the study by Muller et al. used female Wistar rats, whereas we used male C57BL/6 mice, and the toxicological responses to MWCNTs could vary among species and sex.

In the present study, ICP-MS analysis indicates that residual metals were greatly reduced by either thermal or chemical purification. For example, the parent NC7000 sample contained 4.43% aluminum residue which was reduced to 0.04% in the thermally purified MWCNT samples and 0.13% in the chemically purified MWCNT samples. However, since thermally purified derivatives were more toxic than chemically purified derivatives, and both had reduced metals, this suggests that metals were not playing a major role in mediating the pulmonary toxicity of MWCNTs in the present study. Other studies, however, indicated differing conclusions on the role of residual metals in mediating the toxicity of MWCNTs. For example, an early study reported that Fe residues (27% mass of a SWCNT sample) largely contributed to the cytotoxic activity of the SWCNT sample towards macrophages [55] and to the dermal toxicity in mice [56]. In contrast, Co and Fe catalyst residues (< 2% in mass) did not significantly contribute to the acute lung toxicity and genotoxicity of MWCNTs in rat lungs [54]. The apparent discrepancy between these studies might be due to the fact that the first investigators used CNT samples with large amounts of bioavailable metals which can drive Fenton-like reactions to produce reactive oxygen radicals, while in the other study, the metals were almost fully encapsulated within the tubes and thus could not contribute to toxicity. Ni residues (2.2%) have also been reported to contribute to the toxicity of CNTs in vitro and in vivo [57]. The surface properties of engineered nanomaterials in general appear to greatly determine their toxicity, but for CNTs it remains unclear how they exactly do, because of conflicting experimental data. Surface oxidation of MWCNTs by acid treatment increased cytotoxic activity [58, 59]. However, acid treatment of MWCNTs has also been shown to partially remove residual metal (Ni) catalyst and reduce lung inflammation in mice [46]. The MWCNTs used in our study primarily contained aluminum, with some residual cobalt and iron, and depletion of these metals did not reduce pulmonary toxicity of thermally purified MWCNTs. While metal content did not appear to be an important determinant of pulmonary toxicity in the present study, the role of metals in mediating MWCNT-induced toxicity may depend on the specific type of metal catalyst present within the MWCNT structure or whether metals are bioavailable at the surface of MWCNTs.

Several cytokines that were assayed in the BALF of mice from this study (IL-1β, IL-6, and CXCL1) are indicative of acute inflammation and function in the initiation and resolution of neutrophilic inflammation [60]. All three of these cytokines were induced by chemically purified MWCNTs or derivatives at 3 days post-exposure, but not by thermally purified MWCNTs (Fig. 4). Conversely, OPN, a pro-fibrotic cytokine [61], was highly induced by thermally purified MWCNTs and its functionalized derivatives, but not by chemically purified MWCNTs or its functionalized derivatives (Fig. 5). CCL2, a monocyte chemoattractant that plays a role in fibrogenesis [61], was also induced by thermally purified MWCNTs, but not by its functionalized derivatives (Fig. 5). TGF-β1, which plays a major role in fibrogenesis by stimulating extracellular matrix production [61], was not significantly different between groups of mice treated with thermally vs chemically purified MWCNTs. LDH and total protein in BALF were increased chronically at 60 days by thermally purified MWCNTs and its functionalized derivatives, but not by chemically purified MWCNTs or its functionalized derivatives (Fig. 4). Collectively, LDH, total protein, and pro-fibrotic cytokines (CCL2 and OPN) correlated with the number of MWCNT-induced granulomatous lesions in the lungs of mice (Fig. 6). While both thermally and chemically purified MWCNTs induced a similar number of granulomatous lesions, only the thermally purified MWCNTs that were subsequently functionalized with either carboxyl or amine groups induced lesions (Fig. 6). These data indicate that both purification and functionalization are important for the chronic pathological outcome.

Varying the physicochemical properties of CNT can influence toxicity [14, 62, 63]. In the present study, Mitsui-7 MWCNTs were used as a positive control, due to their rigidity and long length [64, 65]. In the mice, exposure to Mitsui-7 induced greater lung infiltration with inflammatory cells, enhanced pro-inflammatory/pro-fibrotic cytokines and induced greater granulomatous lung lesions, when compared to the parent NC7000 MWCNTs. NC7000s were also thinner with a mean diameter of 11.8 nm, whereas Mitsui-7 had a diameter between 49 and 100 nm. MWCNT diameter also seems to determine biological responses. Fenoglio et al. (2012) compared two batches of MWCNTs with identical characteristics except the diameter (10 or 70 nm) and showed that thin MWCNTs were more toxic than thick ones both in vitro (macrophages) and in vivo (rat lung) [66]. Other investigators came to opposite conclusions, but their results might have been confounded by other characteristics of the MWCNTs (e.g. length), which were not well controlled [67–69]. Morphology is also a crucial parameter determining the biological activity of CNTs. While thin MWCNTs (≤ 20 nm diameter) tend to display a tangled morphology with low fiber-specific toxicity, thicker MWCNTs (≥ 30 to < 100 nm) show higher stability and rigidity characterized by a stronger potential to induce mesothelioma or fibrosis in rodents [65, 70, 71]. While rigidity is an important determinant of pulmonary toxicity, it appears that tangled MWCNTs, such as NC7000 used as the parent compound in the present study, are more representative of MWCNTs that are widely used in industry. Therefore, it is important to understand how derivatization of tangled MWCNTs affects toxicity since these are more likely to be associated with human exposure.

A general limitation of most of the above studies is that the experimental samples used were rarely specifically prepared to test a given hypothesis. The MWCNTs that were compared in most of these studies varied with regards to several parameters, thus hampering a formal conclusion on the key physicochemical determinant(s) of toxicity. For instance, investigators who explored the role of length realized that MWCNT shortening was associated with increased surface modifications which contributed to toxicity [72]. In addition, before being functionalized, MWCNTs often need to be oxidized to create sites for covalent reactions; hence it is difficult to discriminate the impact resulting from oxidation from the impact of functionalization. Finally, it should be realized that the critical physicochemical properties may vary depending on the toxicological endpoint considered (e.g. surface defects could drive inflammation but not fibrosis). Therefore, a more systematic approach of the determinants of MWCNT toxicity is therefore strongly needed. The present study represents such an approach using MWCNT derivatives from a common parent compound.

Concern about the health hazards of CNTs requires efforts in basic research to elucidate the mechanistic basis and the physicochemical properties influencing their toxicity. Existing studies, assessing the role of physicochemical properties on CNT toxicity, often yielded contradicting results, possibly because several properties had been changed simultaneously or because different test systems and toxicity endpoints had been evaluated. For these reasons, proper comparison of these studies is nearly impossible. Furthermore, researchers often only reported the physicochemical property that was believed to be important for their specific focus, leaving a substantial uncertainty on other characteristics of the sample. For example, MWCNT inhalation studies differ significantly in the dispersion equipment (pressurized air, acoustic feeder, brush generator), measurement and reporting of the aggregate/agglomerate size distribution, the amount and type of metallic impurities, and the presence of carbon impurities. Taking all of these aspects into account, a precise knowledge of the test items (source, manufacturing conditions, and density of surface functionalization groups) and a thorough documentation of the toxicological methods used are strongly required. A great strength of the present study is the focus on comparison of two purification methods and two subsequent functionalization methods, with all modifications starting from one single MWCNT batch (NC7000) that was already thoroughly tested in rodent in vivo studies [37, 38]. Our findings emphasize that the purification method used prior to functionalization is an important determinant of pulmonary toxicity and could explain why previous studies, comparing similar functionalization methods, generated conflicting results.

## Conclusions

**Fig. 7:**
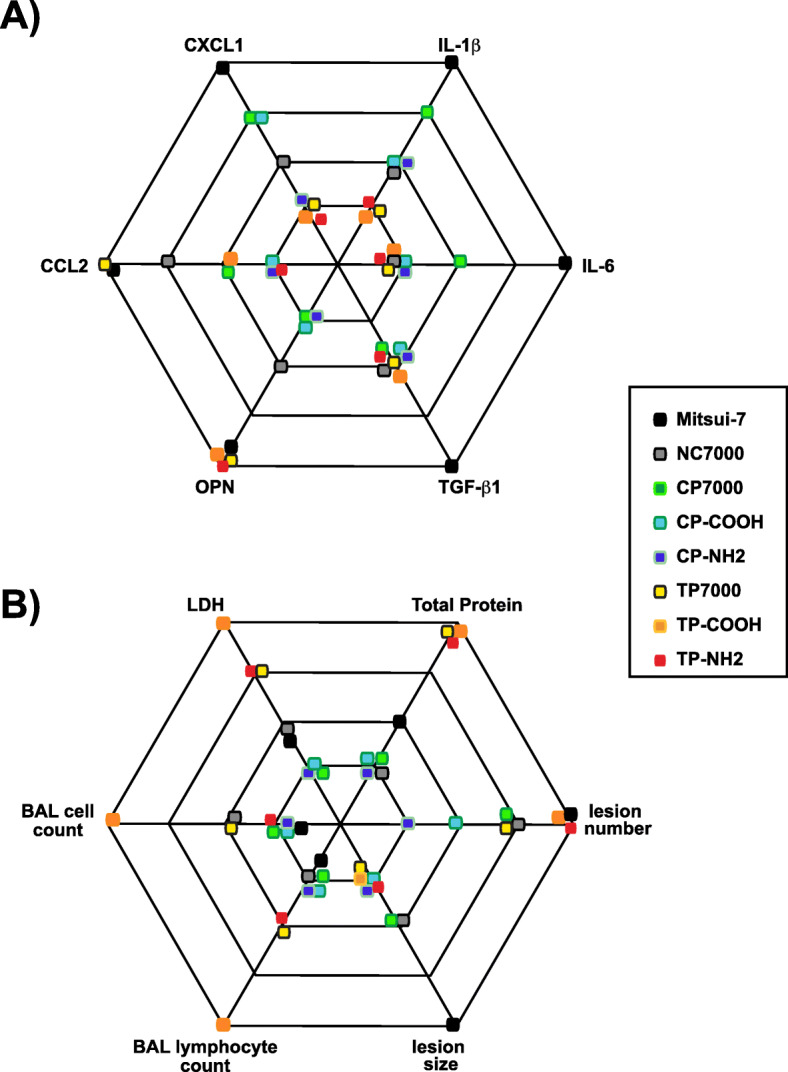
Diagram showing selected biological endpoints at 3 days (**a**) and 60 days (**b**) post-exposure to the different MWCNTs samples used in this study. Increasing distance away from the center indicates increasing biological response. In general, chemically purified MWCNTs and functionalized derivatives caused a greater increase in pro-inflammatory chemokines at 3 days post-exposure, while thermally purified MWCNTs and functionalized derivatives caused a greater increase in pro-fibrotic cytokines (OPN, CCL2) at 3 days and greater pulmonary toxicity (LDH, total protein), BAL lymphocytes, and numbers of granulomas at 60 days post-exposure

This study demonstrates that the purification method is an important determinant of lung inflammation and fibrosis in mice, induced by carboxyl- and amine-functionalized MWCNTs. Thermal purification followed by carboxyl- or amine-functionalization resulted in greater toxicity of MWCNTs, as determined by increased LDH, total protein, OPN and cellularity in BALF at 3 or 60 days post-exposure, as well as increased numbers of granulomas in lung at 60 days post-exposure. As illustrated in Fig. 7, oropharyngeal aspiration of thermally purified MWCNTs or its carboxyl- or amine-functionalized derivatives was associated with greater lung toxicity, increased OPN and CCL2 in BALF, and greater numbers granulomas compared to functionalized derivatives of chemically purified MWCNTs. The greater toxicity of thermally purified derivatives correlated with decreased defects in the carbon framework of MWCNTs, as revealed by Raman spectroscopy. This study provides a deeper understanding of the consequences of different purification methods prior to functionalization that impact the pulmonary toxicity of MWCNTs in mice. Overall, these findings have important implications for assessing the human health risk of MWCNTs in causing lung injury.

## Methods and materials

### MWCNT synthesis, purification and functionalization

All MWCNTs (chemically or thermally purified, carboxylate or amine-functionalized) were custom-synthesized, purified and functionalized by Nanocyl, Inc. (Sambreville, Belgium). They were all generated from a common pristine parent compound, termed NC7000 (Fig. 1a). Thermal purification [heating to 2200–2400 °C] of NC7000 was performed to generate TP7000 MWCNTs. TP7000 was then carboxylated or aminated through a proprietary plasma process to produce TP-COOH and TP-NH2, respectively. Chemical purification [sulfuric acid treatment] of NC7000 was performed to generate CP7000 MWCNTs. CP7000 was then carboxylated or aminated to yield CP-COOH or CP-NH2, respectively. Mitsui-7 MWCNTs (Mitsui & Co., Tokyo, Japan) were obtained from NIOSH, and used as a positive control for induction of fibrosis in the lungs of mice.

### MWCNT characterization

Transmission electron microscopy (TEM) [TEM-FEI Tecnai G20 operating at 200KV] was performed to evaluate length and width of MWCNTs. Electron dispersion X-ray (EDX) spectroscopy (spectrometer attached to a FEI Quanta 3D FEG dual beam microscope operated at 30KV electron voltage) and X-ray photoelectron spectroscopy (XPS) [XPS-SPECS PHOIBOS 150 MCD system equipped with monochromatic Al Kα source (250 W, hν = 1486.6 eV), hemispherical analyzer and multichannel detector] was used to determine elemental composition of the MWCNT samples (carbon, oxygen, nitrogen, aluminum, cobalt and iron). Nitrogen content on the surface of amine functionalized MWCNTs was specifically measured by the Kaiser test [73]. Inductively coupled plasma mass spectrometry (ICP-MS) served to measure the metal content (Al, Co, Fe) of the MWCNT samples. Specific surface area and pore volume of MWCNTs was obtained from N_2_-adsorption–desorption isotherms, using a Sorptomatic 1990 apparatus. The specific surface area was determined by the Brunauer–Emmett–Teller (BET) method, while the pore volumes were measured by the Barret–Joyner–Halenda (BJH) method. Raman spectroscopy was used to quantify the purity and defect density of MWCNTs by assessing the D/G ratio (G-Raman signal given graphite structure/ D-defective structure). The Raman measurements were performed on a confocal Raman microscope (CRM alpha 300R from WiTec GmbH, Germany), using as excitation the 532 nm line of a Nd-YAG laser. The Raman spectra on air-dried droplets were recorded through a 100x objective (NA = 0.9). The integration time was set at 10 s per spectrum. Raman spectra were deconvoluted in the range of 1100–1800 cm^− 1^ into five components following the method reported by Sadezky et al. [39]. A combination of Lorentzian (G, D1, D2, D4 -bands) and Gaussian (D3-band) lines was used, and the intensity of D1 and G was utilized to estimate the ID1/IG ratio (Additional File 2). Agglomeration of the MWCNTs in suspension (hydrodynamic diameter) was evaluated by dynamic light scattering (DLS). Surface charge was evaluated by Zeta potential (Z-potential; agglomerate charge) measurements, using electrophoretic mobility. MWCNT stock suspensions for aspiration exposure were prepared at 2 mg/ml. DLS and Z-potential were determined at a concentration of 50 μg/ml using a Malvern Zetasizer Nano-ZS instrument (Malvern Instruments Ltd., Worcestershire, UK). The measurements were performed at 25 °C, using a 633 nm laser at 90° scattering angle. Samples were equilibrated inside the instrument for 2 min, and five measurements, each consisting of at least twenty runs, were recorded. MWCNTs were tested for endotoxin content to avoid nonspecific pro-inflammatory effects, using the Limulus amoebocyte lysate assay (ThermoFisher, Waltham, MA).

### Animal care

Male C57BL/6 J mice (6–8 weeks old) were purchased from The Jackson Laboratory (Bar Harbor, ME). Mice were housed in an AAALAC (Association for Assessment and Accreditation of Laboratory Animal Care) accredited, pathogen-free animal facility, with controlled humidity and temperature. Mice were allowed to acclimate for at least 2 weeks prior to exposure to MWCNTs. Animals supplied food and water ad libitum. Mice were housed four animals per cage, according to their treatment group. All animal procedures were approved by the NC State University Institutional Animal Care and Committee (IACUC).

### Exposure of mice to MWCNTs

MWCNTs were suspended in 0.1% pluronic F-68 Solution + 3% BSA in Dulbecco’s Phosphate-Buffered Saline (DPBS; Sigma, Saint Louis, MO) and sonicated in a cup horn sonicator (Q500, Qsonica, Newtown, CT) for 30 min at 60 amps, and vortexed immediately before delivery to mice. Mice were exposed to the MWCNTs by oropharyngeal aspiration (OPA) under isoflurane anesthesia to a low (1.6 mg/kg) or high dose (4 mg/kg). The high dose has been used in previous studies [21, 26]. The vehicle (0.1% pluronic + 3% BSA in DPBS) served as negative control and Mitsui-7 (4 mg/kg) as positive control. Due to the number of animals, each time point was split into two groups, both containing negative and positive controls, as well as the 4 mg/kg dose of NC7000. All mice survived the experiment. Necropsy was performed at days 3 and 60 post-exposure.

### Necropsy and sample collection

At necropsy, mice were euthanized with an i.p. injection of pentobarbital. Bronchoalveolar lavage fluid (BALF) was collected by cannulating the trachea and instilling 0.5 ml of DPBS twice. BALF was subsequently used to analyze inflammatory cells, cytokines, lactate dehydrogenase (LDH) activity and total protein. For histopathology, the left lung lobe was fixed by inflation with neutral buffered formalin (VWR, Radnor, PA) for 24 h, then transferred to 70% ethanol for 3 days before paraffin embedding. For mRNA analysis, the right lung lobe was stored in “RNAlater” (Fisher Scientific, Waltham, MA).

### BALF inflammatory cell differentials

For evaluation of lung inflammatory cells, 100 μl of BALF was centrifuged using a Cytospin 4 centrifuge (ThermoFisher, Waltham, MA) to isolate cells on glass slides. The slides were then fixed and stained with the “Diff-Quik” stain set (Siemens, Newark, DE). The average count of all cell types was quantified by using an Olympus light microscope BX41 (Center Valley, PA) with three representative photomicrographs taken per animal at 100 x magnification, and every cell type counted using ImageJ software with Fiji expansion [Eliceiri/LOCI group, University of Wisconsin-Madison, Madison, WI). This method has been validated and published previously [25]. Cell differentials were quantified by counting 500 cells per slide/animal to determine relative numbers of macrophages, neutrophils, eosinophils and lymphocytes.

### Cytotoxicity and total protein in BALF

LDH activity in BALF was assayed as an indicator for pulmonary cytotoxicity with the “Pierce LDH Cytotoxicity Assay Kit” (ThermoFisher, Waltham, MA), according to the manufacturer’s instructions. Absorbance values were finally measured at 450 nm using a Multiskan EX microplate spectrophotometer (ThermoFisher, Waltham, MA). Total protein concentration in BALF was determined, according to the manufacturer’s protocol with the “Pierce BCA Protein Assay Kit” (ThermoFisher, Waltham, MA). Absorbance was finally read at 450 nm with a background correction at 540 nm, using a Multiskan EX microplate spectrophotometer (ThermoFisher, Waltham, MA).

### Cytokine quantification in BALF

Cytokine-specific DuoSet enzyme-linked immunosorbent assay (ELISA) kits (R&D Systems, Minneapolis, MN) were used according to the manufacturer’s protocol to quantify protein levels of cytokines (i.e., OPN, TGF- β1, CCL2, IL-1β, CXCL1, IL-6) in BALF. Values were expressed as fold change instead of pg/ml due to the experiment having two groups per time point.

### Histopathology and morphometry

The left lung lobe was cut into three sections and embedded in paraffin. Tissue sections on glass slides were stained with Gomori’s trichrome staining was done to assess collagen deposition. Gomori’s trichrome-stained slides were analyzed by light microscopy to determine the area (μm^2^) and total number of granulomatous lung lesions containing MWCNTs at day 60 post-exposure in each mouse lung (3 lung cross-sections per mouse). To determine the average area of the lesions, approximately 30 photomicrographs per mouse lung were taken and analyzed in Photoshop CS5 (Adobe, San Jose, CA), using the lasso measurement tool. 

### Statistical analysis

One-way ANOVA with a Tukey post-hoc test was utilized to evaluate differences between treatment groups (GraphPad Prism, version 5.0, La Jolla, CA). Comparisons were made between all MWCNTs relative to vehicle control, between NC7000 and purified forms (TP7000 and CP7000), and between carboxyl- or amine-functionalized forms and the relevant purified parent MWCNT samples.

## Supplementary Information

**Additional file 1.** Raman spectra of MWCNTs samples showing the G (~ 1570 cm^− 1^) and D (~ 1346 cm^− 1^) mode.

**Additional file 2.** Curve fitting of first ordered Raman spectra.

**Additional file 3.** Estimation of ID1/IG ratios from Raman spectroscopy data.

**Additional file 4. **XPS C1s High resolution spectra with the peak positions of the possible bonds*. (PDF 176 kb)*

## Data Availability

Datasets generated for this study are available from the corresponding author upon reasonable request.

## Abbreviations

ALD: Atomic layer deposition
BALF: Bronchoalveolar lavage fluid
BET: Brunauer–Emmett–Teller
BSA: Bovine serum albumin
CCL2: CC chemokine ligand 2
CXCL1: CXC chemokine 1
CNT: Carbon nanotube
DPBS: Dulbecco’s phosphate buffered saline
DLS: Dynamic light scattering
EDX: Electron dispersion X-ray spectroscopy
ICP-MS: Inductively coupled plasma mass spectrometry
IL: Interleukin
LDH: Lactate dehydrogenase
OPN: Osteopontin
OPA: Oropharyngeal aspiration
pDi: Polydispersity index
PC: Physicochemical
TEM: Transmission electron microscopy
TGF-β1: Transforming growth factor-β1
MWCNT: Multiwalled carbon nanotube
XPS: X-ray photoelectron (XPS) spectroscopy

## References

[1] Iijima S (1991). Helical microtubules of graphitic carbon. Nature.

[2] Frank S, Poncharal P, Wang ZL, de WA H (1998). Carbon Nanotube Quantum Resistors. Science.

[3] Wong EW, Sheehan PE, Lieber CM (1997). Nanobeam Mechanics: Elasticity, Strength, and Toughness of Nanorods and Nanotubes. Science.

[4] Liu Z, Tabakman S, Welsher K, Dai H (2009). Carbon nanotubes in biology and medicine: in vitro and in vivo detection, imaging and drug delivery. Nano Res.

[5] Pacurari M, Lowe K, Tchounwou PB, Kafoury R (2016). A review on the respiratory system toxicity of carbon nanoparticles. Int J Environ Res Public Health.

[6] Maynard AD, Baron PA, Foley M, Shvedova AA, Kisin ER, Castranova V (2004). Exposure to carbon nanotube material: aerosol release during the handling of unrefined single-walled carbon nanotube material. J Toxicol Environ Heal Part A.

[7] Han JH, Lee EJ, Lee JH, So KP, Lee YH, Bae GN (2008). Monitoring multiwalled carbon nanotube exposure in carbon nanotube research facility. Inhal Toxicol.

[8] Lee JH, Lee S-B, Bae GN, Jeon KS, Yoon JU, Ji JH (2010). Exposure assessment of carbon nanotube manufacturing workplaces. Inhal Toxicol.

[9] Dahm MM, Schubauer-Berigan MK, Evans DE, Birch ME, Fernback JE, Deddens JA (2015). Carbon nanotube and Nanofiber exposure assessments: an analysis of 14 site visits. Ann Occup Hyg..

[10] Kuijpers E, Bekker C, Fransman W, Brouwer D, Tromp P, Vlaanderen J (2015). Occupational exposure to multi-walled carbon nanotubes during commercial production synthesis and handling. Ann Occup Hyg.

[11] Shvedova AA, Yanamala N, Kisin ER, Birch ME, Fatkhutdinova LM, Khailullin TO (2016). Integrated analysis of Dysregulated ncRNA and mRNA expression profiles in humans exposed to carbon nanotubes. PLoS One.

[12] Fatkhutdinova LM, Vasil’yeva OL, Zalyalov RR, Mustafin IG, Kisin ER, Khaliullin TO (2016). Fibrosis biomarkers in workers exposed to MWCNTs. Toxicol Appl Pharmacol.

[13] Donaldson K, Aitken R, Tran L, Stone V, Duffin R, Forrest G (2006). Carbon nanotubes: a review of their properties in relation to pulmonary toxicology and workplace safety. Toxicol Sci.

[14] Duke KS, Bonner JC (2018). Mechanisms of carbon nanotube-induced pulmonary fibrosis: a physicochemical characteristic perspective. WIREs Nanomedicine and Nanobiotechnology.

[15] Shvedova AA, Pietroiusti A, Fadeel B, Kagan VE (2012). Mechanisms of carbon nanotube-induced toxicity: focus on oxidative stress. Toxicol Appl Pharmacol.

[16] Donaldson K, Poland CA, Murphy FA, MacFarlane M, Chernova T, Schinwald A (2013). Pulmonary toxicity of carbon nanotubes and asbestos — similarities and differences. Adv Drug Deliv Rev.

[17] Port J, Murphy DJ (2017). Mesothelioma: identical routes to malignancy from Asbestos and carbon nanotubes. Curr Biol.

[18] Chernova T, Murphy FA, Galavotti S, Sun X-M, Powley IR, Grosso S (2017). Long-Fiber Carbon Nanotubes Replicate Asbestos-Induced Mesothelioma with Disruption of the Tumor Suppressor Gene Cdkn2a (Ink4a/Arf). Curr Biol.

[19] Dymacek JM, Snyder-Talkington BN, Raese R, Dong C, Singh S, Porter DW (2018). Similar and differential canonical pathways and biological processes associated with multiwalled carbon nanotube and Asbestos-induced pulmonary fibrosis: a 1-year Postexposure study. Int J Toxicol.

[20] Boyles MSP, Young L, Brown DM, MacCalman L, Cowie H, Moisala A (2015). Multi-walled carbon nanotube induced frustrated phagocytosis, cytotoxicity and pro-inflammatory conditions in macrophages are length dependent and greater than that of asbestos. Toxicol Vitr.

[21] Porter DW, Hubbs AF, Mercer RR, Wu N, Wolfarth MG, Sriram K, et al. Mouse pulmonary dose- and time course-responses induced by exposure to multi-walled carbon nanotubes. Toxicology. 269:136–47. 10.1016/j.tox.2009.10.017. 10.1016/j.tox.2009.10.017 19857541

[22] Sargent LM, Porter DW, Staska LM, Hubbs AF, Lowry DT, Battelli L (2014). Promotion of lung adenocarcinoma following inhalation exposure to multi-walled carbon nanotubes. Part Fibre Toxicol..

[23] Ryman-Rasmussen JP, Tewksbury EW, Moss OR, Cesta MF, Wong BA, Bonner JC (2009). Inhaled multiwalled carbon nanotubes potentiate airway fibrosis in murine allergic asthma. Am J Respir Cell Mol Biol.

[24] Ihrie MD, Taylor-Just AJ, Walker NJ, Stout MD, Gupta A, Richey JS (2019). Inhalation exposure to multi-walled carbon nanotubes alters the pulmonary allergic response of mice to house dust mite allergen. Inhal Toxicol.

[25] Shipkowski KA, Taylor AJ, Thompson EA, Glista-Baker EE, Sayers BC, Messenger ZJ (2015). An allergic lung microenvironment suppresses carbon nanotube-induced Inflammasome activation via STAT6-dependent inhibition of Caspase-1. PLoS One.

[26] Thompson EA, Sayers BC, Glista-Baker EE, Shipkowski KA, Ihrie MD, Duke KS (2015). Role of signal transducer and activator of transcription 1 in murine allergen–induced airway remodeling and exacerbation by carbon nanotubes. Am J Respir Cell Mol Biol.

[27] Pacurari M, Castranova V, Vallyathan V (2010). Single- and Multi-Wall carbon nanotubes versus Asbestos: are the carbon nanotubes a new health risk to humans?. J Toxicol Environ Heal Part A..

[28] De Volder MFL, Tawfick SH, Baughman RH, Hart AJ (2013). Carbon Nanotubes: Present and Future Commercial Applications. Science.

[29] Porter DW, Orandle M, Zheng P, Wu N, Hamilton RF, Holian A (2020). Mouse pulmonary dose- and time course-responses induced by exposure to nitrogen-doped multi-walled carbon nanotubes. Inhal Toxicol.

[30] Poulsen SS, Jackson P, Kling K, Knudsen KB, Skaug V, Kyjovska ZO (2016). Multi-walled carbon nanotube physicochemical properties predict pulmonary inflammation and genotoxicity. Nanotoxicology..

[31] Sager TM, Wolfarth MW, Andrew M, Hubbs A, Friend S, Chen T (2014). Effect of multi-walled carbon nanotube surface modification on bioactivity in the C57BL/6 mouse model. Nanotoxicology..

[32] Li R, Wang X, Ji Z, Sun B, Zhang H, Chang CH (2013). Surface charge and cellular processing of covalently functionalized multiwall carbon nanotubes determine pulmonary toxicity. ACS Nano.

[33] Tong H, McGee JK, Saxena RK, Kodavanti UP, Devlin RB, Gilmour MI (2009). Influence of acid functionalization on the cardiopulmonary toxicity of carbon nanotubes and carbon black particles in mice. Toxicol Appl Pharmacol.

[34] Dandley EC, Taylor AJ, Duke KS, Ihrie MD, Shipkowski KA, Parsons GN (2016). Atomic layer deposition coating of carbon nanotubes with zinc oxide causes acute phase immune responses in human monocytes in vitro and in mice after pulmonary exposure. Part Fibre Toxicol.

[35] Taylor AJ, McClure CD, Shipkowski KA, Thompson EA, Hussain S, Garantziotis S (2014). Atomic layer deposition coating of carbon nanotubes with aluminum oxide alters pro-fibrogenic cytokine expression by human mononuclear phagocytes in vitro and reduces lung fibrosis in mice in vivo. PLoS One.

[36] Zhou L, Forman HJ, Ge Y, Lunec J (2017). Multi-walled carbon nanotubes: a cytotoxicity study in relation to functionalization, dose and dispersion. Toxicol in Vitro.

[37] Ma-Hock L, Treumann S, Strauss V, Brill S, Luizi F, Mertler M (2009). Inhalation toxicity of multiwall carbon nanotubes in rats exposed for 3 months. Toxicol Sci.

[38] Treumann S, Ma-Hock L, Gröters S, Landsiedel R, van Ravenzwaay B (2013). Additional Histopathologic examination of the lungs from a 3-month inhalation toxicity study with multiwall carbon nanotubes in rats. Toxicol Sci.

[39] Sadezky A, Muckenhuber H, Grothe H, Niessner R, Poschl U (2005). Raman microspectroscopy of soot and related carbonaceous materials: spectral analysis and structural information. Carbon.

[40] Lehman JH, Terrones M, Mansfield E, Hurst KE, Meunier V (2011). Evaluating the characteristics of multiwall carbon nanotubes. Carbon.

[41] DiLeo RA, Landi BJ, Raffaelle R (2007). Purity assessment of multiwalled carbon nanotubes by Raman spectroscopy. J Appl Phys.

[42] Firme CP, Bandaru PR (2010). Toxicity issues in the application of carbon nanotubes to biological systems. Nanomedicine Nanotechnology.

[43] Sayes CM, Liang F, Hudson JL, Mendez J, Guo W, Beach JM (2006). Functionalization density dependence of single-walled carbon nanotubes cytotoxicity in vitro. Toxicol Lett.

[44] Roda E, Coccini T, Acerbi D, Barni S, Vaccarone R, Manzo L. Comparative pulmonary toxicity assessmentof pristine and functionalized Multi-WalledCarbon Nanotubes intratracheally instilledin rats: morphohistochemical evaluations. Histol Histopathol Vol 26, n^o^ 3. Murcia: F. Hernández; 2011; https://digitum.um.es/digitum/handle/10201/48286. 10.14670/HH-26.357 21210349

[45] Hamilton RF, Xiang C, Li M, Ka I, Yang F, Ma D (2013). Purification and sidewall functionalization of multiwalled carbon nanotubes and resulting bioactivity in two macrophage models. Inhal Toxicol.

[46] Bonner JC, Silva RM, Taylor AJ, Brown JM, Hilderbrand SC, Castranova V (2013). Interlaboratory evaluation of rodent pulmonary responses to engineered nanomaterials: the NIEHS Nano GO consortium. Environ Health Perspect.

[47] Wang X, Xia T, Ntim SA, Ji Z, George S, Meng H (2010). Quantitative techniques for assessing and controlling the dispersion and biological effects of multiwalled carbon nanotubes in mammalian tissue culture cells. ACS Nano.

[48] Wang X, Xia T, Addo Ntim S, Ji Z, Lin S, Meng H (2011). Dispersal state of multiwalled carbon nanotubes elicits Profibrogenic cellular responses that correlate with Fibrogenesis biomarkers and fibrosis in the murine lung. ACS Nano.

[49] Poland CA, Duffin R, Kinloch I, Maynard A, Wallace WAH, Seaton A (2008). Carbon nanotubes introduced into the abdominal cavity of mice show asbestos-like pathogenicity in a pilot study. Nat Nanotechnol.

[50] Ali-Boucetta H, Nunes A, Sainz R, Herrero MA, Tian B, Prato M (2013). Asbestos-like pathogenicity of long carbon nanotubes alleviated by chemical functionalization. Angew Chemie Int Ed.

[51] Wepasnick KA, Smith BA, Bitter JL, Fairbrother DH (2010). Chemical and structural characterization of carbon nanotube surfaces. Anal Bioanal Chem.

[52] Rebelo SLH, Guedes A, Szefczyk ME, Pereira AM, Araujo JP, Freire C (2016). Progress in the Raman spectra analysis of covalently functionalized multiwalled carbon nanotubes: unraveling disorder in graphitic materials. Phys Chem Chem Phys.

[53] Schönfelder R, Avilés F, Knupfer M, Azamar-Barrios JA, González-Chi PI, Rümmeli MH. Influence of architecture on the Raman spectra of acid treated carbon nanostructures. J Exp Nanoscience. 9:931–41. 10.1080/17458080.2012.750763.

[54] Muller J, Huaux F, Fonseca A, Nagy JB, Moreau N, Delos M (2008). Structural defects play a major role in the acute lung toxicity of multiwall carbon nanotubes: toxicological aspects. Chem Res Toxicol.

[55] Kagan VE, Tyurina YY, Tyurin VA, Konduru NV, Potapovich AI, Osipov AN (2006). Direct and indirect effects of single walled carbon nanotubes on RAW 264.7 macrophages: Role of iron. Toxicol Lett.

[56] Murray AR, Kisin E, Leonard SS, Young SH, Kommineni C, Kagan VE (2009). Oxidative stress and inflammatory response in dermal toxicity of single-walled carbon nanotubes. Toxicology..

[57] Hamilton RF, Buford M, Xiang C, Wu N, Holian A (2012). NLRP3 inflammasome activation in murine alveolar macrophages and related lung pathology is associated with MWCNT nickel contamination. Inhal Toxicol.

[58] Bottini M, Bruckner S, Nika K, Bottini N, Bellucci S, Magrini A (2006). Multi-walled carbon nanotubes induce T lymphocyte apoptosis. Toxicol Lett.

[59] Vittorio O, Raffa V, Cuschieri A (2009). Influence of purity and surface oxidation on cytotoxicity of multiwalled carbon nanotubes with human neuroblastoma cells. Nanomedicine Nanotechnology, Biol Med.

[60] You DJ, Lee HY, Taylor-Just AJ, Linder KE, Bonner JC (2020). Sex differences in the acute and subchronic lung inflammatory responses of mice to nickel nanoparticles. Nanotoxicology..

[61] Bonner JC (2010). Mesenchymal cell survival in airway and interstitial pulmonary fibrosis. Fibrogenesis Tissue Repair.

[62] Johnston HJ, Hutchison G, Christensen FM, Peters S, Hankin S, Stone V (2010). A review of the in vivo and in vitro toxicity of silver and gold particulates: particle attributes and biological mechanisms responsible for the observed toxicity. Crit Rev Toxicol.

[63] Lanone S, Andujar P, Kermanizadeh A, Boczkowski J (2013). Determinants of carbon nanotube toxicity. Adv Drug Deliv Rev.

[64] Rahman L, Jacobsen NR, Aziz SA, Wu D, Williams A, Yauk CL (2017). Multi-walled carbon nanotube-induced genotoxic, inflammatory and pro-fibrotic responses in mice: investigating the mechanisms of pulmonary carcinogenesis. Mutat Res Toxicol Environ Mutagen.

[65] Duke KS, Thompson EA, Ihrie MD, Taylor-Just AJ, Ash EA, Shipkowski KA (2018). Role of p53 in the chronic pulmonary immune response to tangled or rod-like multi-walled carbon nanotubes. Nanotoxicology. 2018/10/14.

[66] Fenoglio I, Aldieri E, Gazzano E, Cesano F, Colonna M, Scarano D (2012). Thickness of multiwalled carbon nanotubes affects their lung toxicity. Chem Res Toxicol.

[67] Nagai H, Okazaki Y, Chew SH, Misawa N, Yamashita Y, Akatsuka S (2011). Diameter and rigidity of multiwalled carbon nanotubes are critical factors in mesothelial injury and carcinogenesis. Proc Natl Acad Sci U S A.

[68] Wang X, Jia G, Wang H, Nie H, Yan L, Deng XY (2009). Diameter effects on cytotoxicity of multi-walled carbon nanotubes. J Nanosci Nanotechnol.

[69] Yamashita K, Yoshioka Y, Higashisaka K, Morishita Y, Yoshida T, Fujimura M (2010). Carbon nanotubes elicit DNA damage and inflammatory response relative to their size and shape. Inflammation..

[70] Rittinghausen S, Hackbarth A, Creutzenberg O, Ernst H, Heinrich U, Leonhardt A (2014). The carcinogenic effect of various multi-walled carbon nanotubes (MWCNTs) after intraperitoneal injection in rats. Part Fibre Toxicol..

[71] Mercer RR, Scabilloni JF, Hubbs AF, Battelli LA, McKinney W, Friend S (2013). Distribution and fibrotic response following inhalation exposure to multi-walled carbon nanotubes. Part Fibre Toxicol..

[72] Bussy C, Pinault M, Cambedouzou J, Landry MJ, Jegou P, Mayne-L’hermite M (2012). Critical role of surface chemical modifications induced by length shortening on multi-walled carbon nanotubes-induced toxicity. Part Fibre Toxicol..

[73] Sarin VK, Kent SBH, Tam JP, Merrifield RB (1981). Quantitative monitoring of solid-phase peptide synthesis by the ninhydrin reaction. Anal Biochem.

