# Supplementary material for: The pulmonary toxicity of carboxylated or aminated multi-walled carbon nanotubes in mice is determined by the prior purification method
Source: Part Fibre Toxicol. 2020 Nov 26;17:60. doi: 10.1186/s12989-020-00390-y (PMC7690083; doi:10.1186/s12989-020-00390-y)
Supplement: Supplementary file 1 — Additional file 1. Raman spectra of MWCNTs samples showing the G (~ 1570 cm− 1) and D (~ 1346 cm− 1) mode. [file 12989_2020_390_MOESM1_ESM.pdf]

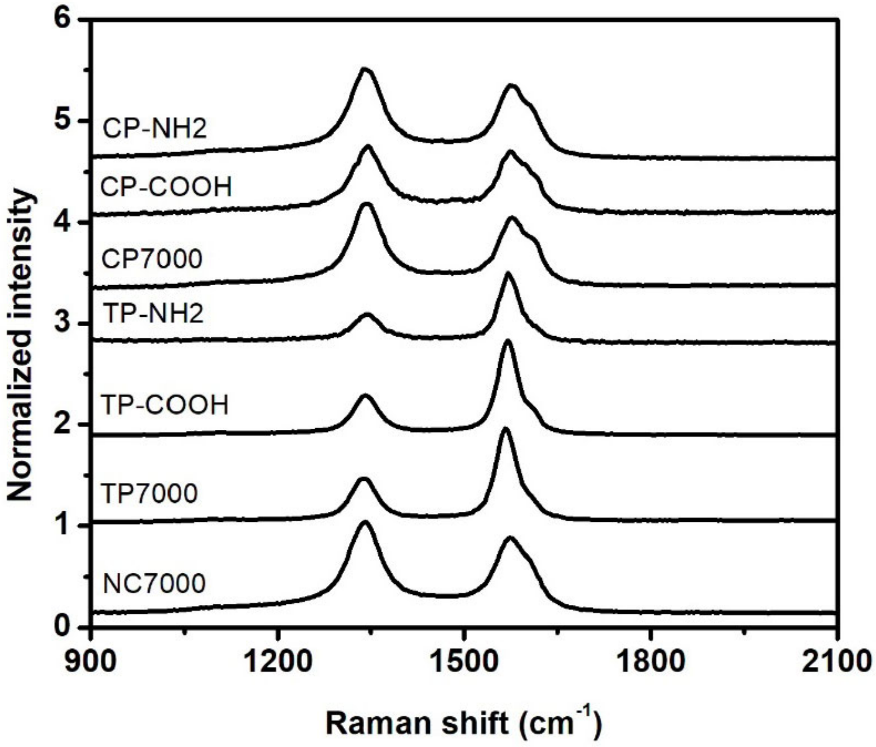

Additional File 1. Raman spectra of MWCNTs samples showing the G (~1570 cm^{-1}) and D (~1346 cm^{-1}) mode. The spectra were normalized on the most intense band.
