# Supplementary material for: The pulmonary toxicity of carboxylated or aminated multi-walled carbon nanotubes in mice is determined by the prior purification method
Source: Part Fibre Toxicol. 2020 Nov 26;17:60. doi: 10.1186/s12989-020-00390-y (PMC7690083; doi:10.1186/s12989-020-00390-y)
Supplement: Supplementary file 2 — Additional file 2. Curve fitting of first ordered Raman spectra. [file 12989_2020_390_MOESM2_ESM.pdf]

Additional File 2

Curve fitting of first ordered Raman spectra

Band	Raman shift (cm $^{-1}$)	Vibration mode 10.1016/j.carbon.2005.02.018
G	~1580	Ideal graphitic lattice
D1	~1350	Disordered graphitic lattice (graphene layer edges),
D2(D')	~1620	Disordered graphitic lattice (surface graphene layers)
D3	~1500	Amorphous carbon
D4	~1200	Disordered graphitic lattice , polyenes, ionic impurities

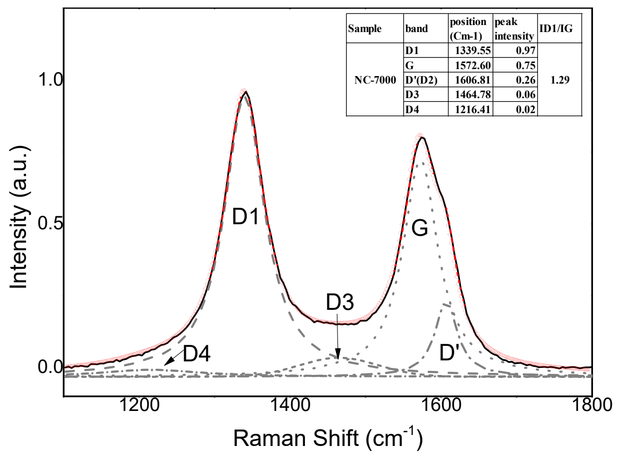

Sample	band	position (Cm-1)	peak intensity	ID1/IG
NC-7000	D1	1339.55	0.97	1.29
	G	1572.60	0.75	
	D'(D2)	1606.81	0.26	
	D3	1464.78	0.06	
	D4	1216.41	0.02	

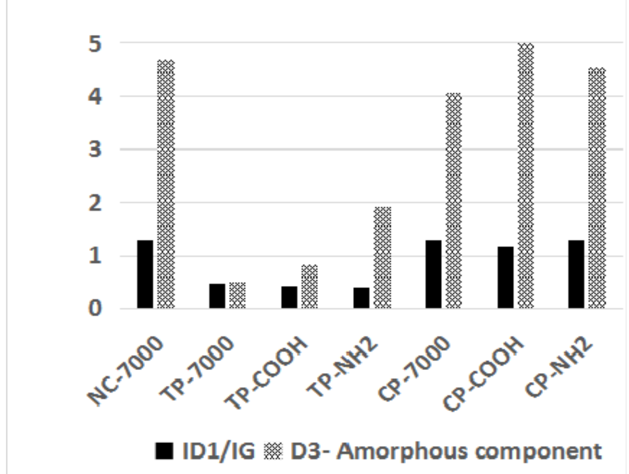

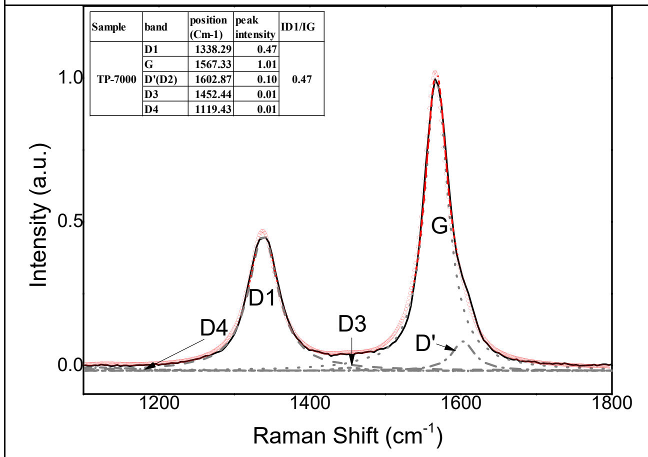

Sample	band	position (Cm-1)	peak intensity	ID1/IG
TP-7000	D1	1338.29	0.47	0.47
	G	1567.33	1.01	
	D'(D2)	1602.87	0.10	
	D3	1452.44	0.01	
	D4	1119.43	0.01	

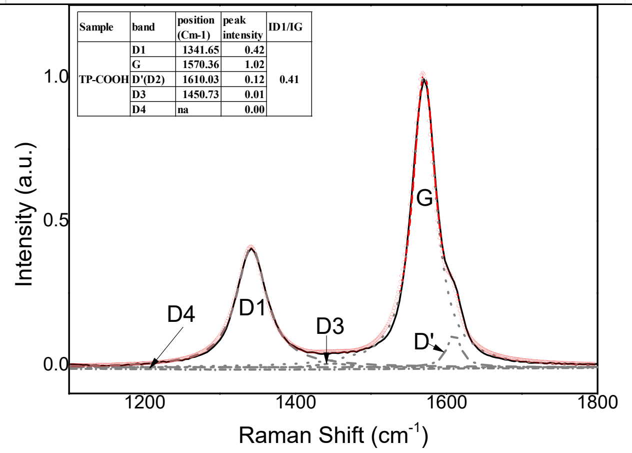

Sample	band	position (Cm-1)	peak intensity	ID1/IG
TP-COOH	D1	1341.65	0.42	0.41
	G	1570.36	1.02	
	D'(D2)	1610.03	0.12	
	D3	1450.73	0.01	
	D4	na	0.00	

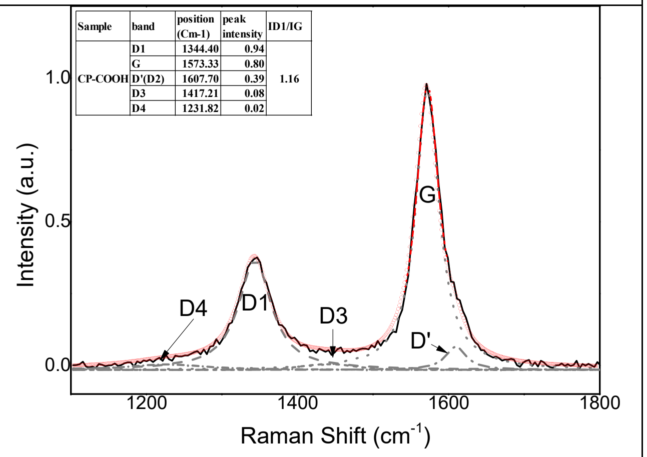

Sample	band	position (Cm-1)	peak intensity	ID1/IG
CP-COOH	D1	1344.40	0.94	1.16
	G	1573.33	0.80	
	D'(D2)	1607.70	0.39	
	D3	1417.21	0.08	
	D4	1231.82	0.02	

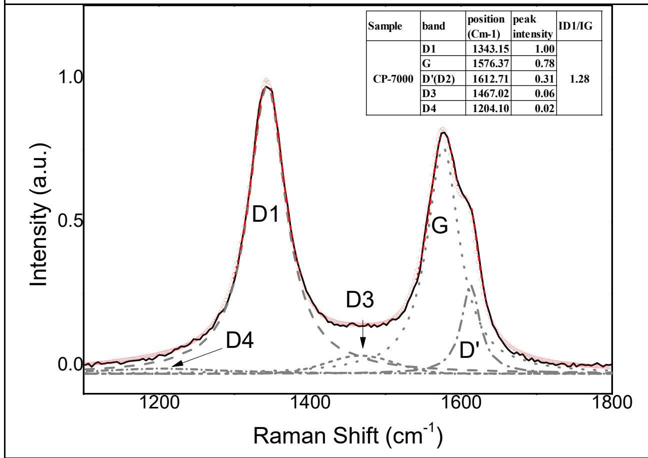

Sample	band	position (Cm-1)	peak intensity	ID1/IG
CP-7000	D1	1343.15	1.00	1.28
	G	1576.37	0.78	
	D'(D2)	1612.71	0.31	
	D3	1467.02	0.06	
	D4	1204.10	0.02	

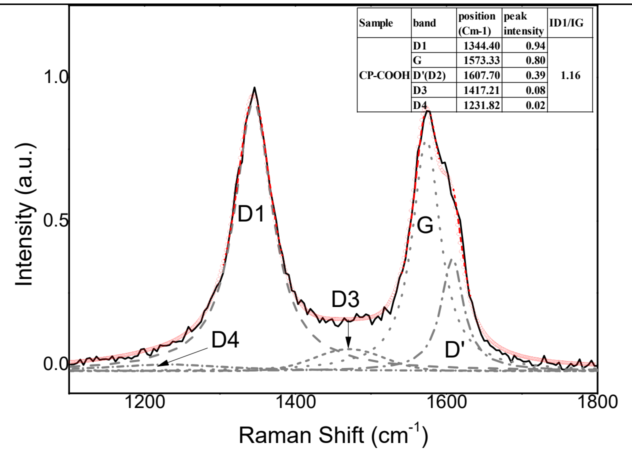

Sample	band	position (Cm-1)	peak intensity	ID1/IG
CP-COOH	D1	1344.40	0.94	1.16
	G	1573.33	0.80	
	D'(D2)	1607.70	0.39	
	D3	1417.21	0.08	
	D4	1231.82	0.02	

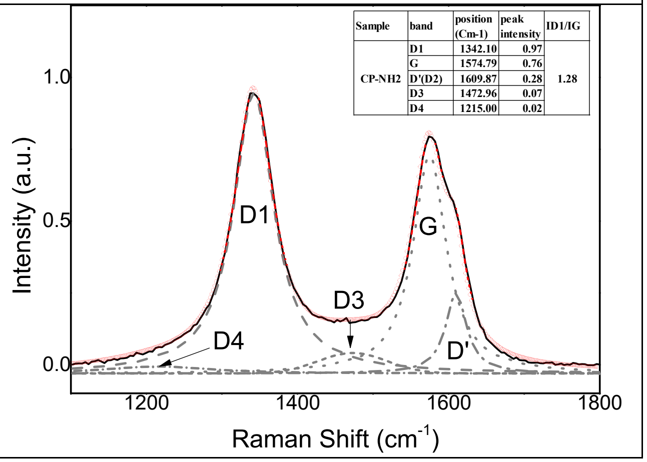

Sample	band	position (Cm-1)	peak intensity	ID1/IG
CP-NH2	D1	1342.10	0.97	1.28
	G	1574.79	0.76	
	D'(D2)	1609.87	0.28	
	D3	1472.96	0.07	
	D4	1215.00	0.02	
