# Supplementary material for: The pulmonary toxicity of carboxylated or aminated multi-walled carbon nanotubes in mice is determined by the prior purification method
Source: Part Fibre Toxicol. 2020 Nov 26;17:60. doi: 10.1186/s12989-020-00390-y (PMC7690083; doi:10.1186/s12989-020-00390-y)
Supplement: Supplementary file 3 — Additional file 3. Estimation of ID1/IG ratios from Raman spectroscopy data. [file 12989_2020_390_MOESM3_ESM.pdf]

Additional File 3. Estimation of ID1/IG ratios from Raman spectroscopy data.

Sample	band	position (Cm^{-1})	peak intensity	peak area	% from total area	ID1/IG
NC-7000	D1	1339.55	0.97	92.42	50.52	1.29
	G	1572.60	0.75	63.12	34.51	
	D'(D2)	1606.81	0.26	14.23	7.78	
	D3	1464.78	0.06	8.57	4.68	
	D4	1216.41	0.02	4.58	2.51	
TP-7000	D1	1338.29	0.47	33.92	34.37	0.47
	G	1567.33	1.01	57.63	58.40	
	D'(D2)	1602.87	0.10	5.50	5.58	
	D3	1452.44	0.01	0.48	0.48	
	D4	1119.43	0.01	1.13	1.15	
TP-COOH	D1	1341.65	0.42	30.41	34.47	0.41
	G	1570.36	1.02	57.28	64.92	
	D'(D2)	1610.03	0.12	3.85	4.37	
	D3	1450.73	0.01	0.72	0.81	
	D4	na	0.00	0.00	0.00	
TP-NH2	D1	1343.60	0.38	29.84	32.03	0.39
	G	1572.04	0.97	54.46	58.45	
	D'(D2)	1609.66	0.08	4.03	4.32	
	D3	1440.27	0.02	1.79	1.92	
	D4	1227.41	0.02	3.05	3.27	
CP-7000	D1	1343.15	1.00	88.38	50.71	1.28
	G	1576.37	0.78	60.94	34.97	
	D'(D2)	1612.71	0.31	13.88	7.97	
	D3	1467.02	0.06	7.08	4.07	
	D4	1204.10	0.02	3.99	2.29	
CP-COOH	D1	1344.40	0.94	84.78	48.54	1.16
	G	1573.33	0.80	54.75	31.35	
	D'(D2)	1607.70	0.39	21.37	12.24	
	D3	1417.21	0.08	8.74	5.00	
	D4	1231.82	0.02	5.02	2.88	
CP-NH2	D1	1342.10	0.97	92.42	51.34	1.28
	G	1574.79	0.76	60.18	33.43	
	D'(D2)	1609.87	0.28	14.28	7.93	
	D3	1472.96	0.07	8.15	4.53	
	D4	1215.00	0.02	4.97	2.76	
