# Supplementary material for: The pulmonary toxicity of carboxylated or aminated multi-walled carbon nanotubes in mice is determined by the prior purification method
Source: Part Fibre Toxicol. 2020 Nov 26;17:60. doi: 10.1186/s12989-020-00390-y (PMC7690083; doi:10.1186/s12989-020-00390-y)
Supplement: Supplementary file 4 — Additional file 4. XPS C1s High resolution spectra with the peak positions of the possible bonds. (PDF 176 kb) [file 12989_2020_390_MOESM4_ESM.pdf]

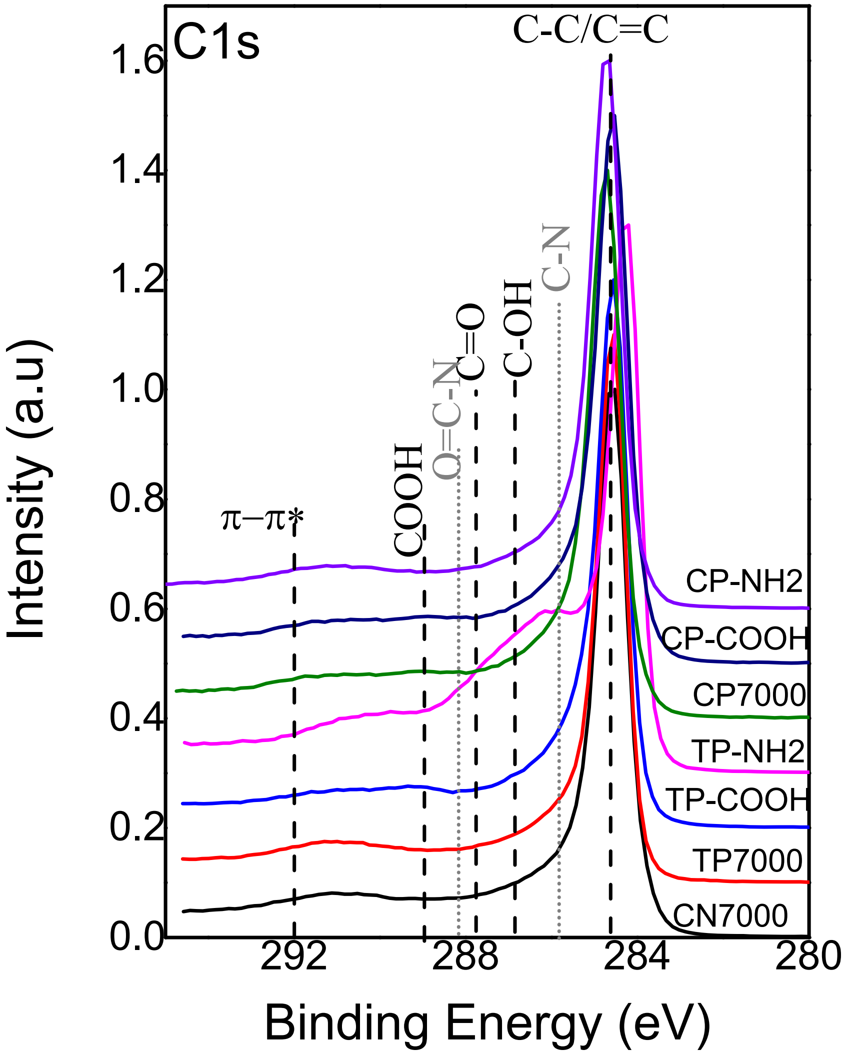

Additional File 4. XPS C1s High resolution spectra with the peak positions of the possible bonds. *(http://dx.doi.org/10.1016/j.bej.2014.05.009)*
